# Topical moisturizer with rose stem cell‐derived exosomes (RSCEs) for recalcitrant seborrheic dermatitis: A case report with 6 months of follow‐up

**DOI:** 10.1111/jocd.16389

**Published:** 2024-06-03

**Authors:** Suparuj Lueangarun, Byong Seung Cho, Therdpong Tempark

**Affiliations:** ^1^ Department of Aesthetic Medicine College of Integrative Medicine, Dhurakij Pundit University Bangkok Thailand; ^2^ Division of Dermatology DeMed Clinic Center Bangkok Thailand; ^3^ ExoCoBio Exosome Institute (EEI) ExoCoBio Inc. Seoul Republic of Korea; ^4^ Department of Pediatrics, Faculty of Medicine, King Chulalongkorn Memorial Hospital Chulalongkorn University Pathum Wan Bangkok Thailand

**Keywords:** exosomes, inflammatory skin condition, moisturizer, seborrheic dermatitis, topical treatment

## Abstract

**Introduction:**

Seborrheic dermatitis (SD) poses significant treatment challenges due to its chronic nature and the side effects associated with long‐term use of conventional therapies like topical corticosteroids. In the search for alternative treatments, exosomes, particularly those derived from rose stem cells (RSCEs), offer a promising avenue due to their potential in managing chronic skin conditions.

**Objective:**

This case report examines the efficacy of a topical moisturizer containing RSCEs in treating a patient with refractory SD, aiming to provide an alternative treatment pathway.

**Materials and Methods:**

A 40‐year‐old male with a long‐standing history of SD, unresponsive to traditional treatments, underwent a novel treatment regimen. This regimen included an initial topical application of 2.5 mL of RSCEs followed by a maintenance phase involving the application of a RSCE‐containing moisturizer. Clinical outcomes were assessed through the Patient's Global Assessment (PGA) and Investigator's Global Assessment (IGA) scores, along with evaluations of scaling and erythema.

**Results:**

Remarkable clinical improvement was noted as early as 1‐day post‐treatment, with significant reductions in redness, scaling, and itching. The patient experienced sustained relief throughout the 6‐month follow‐up, with a recurrence in the sixth month that was less severe than previous flare‐ups. This demonstrated not only the efficacy of RSCEs in symptom management but also their potential in extending remission periods.

**Conclusion:**

The chronic management of SD can benefit from innovative treatments like the RSCE‐containing moisturizer, as shown in this case report. While the observed outcomes are promising, indicating substantial improvements in skin condition and symptom management, larger controlled studies are necessary to validate the therapeutic potential of exosome‐containing moisturizers fully. This case underscores the need for alternative therapies in SD treatment, highlighting the role of exosomes as a viable option.

**Abbreviations**
AD: atopic dermatitis
DFR: dupilumab‐related facial redness
EVs: extracellular vesicles
MSCs: mesenchymal stem cells
RSCEs: rose stem cell‐derived exosomes
SD: seborrheic dermatitis

## 1 INTRODUCTION

Seborrheic dermatitis (SD) is one of the prevalent inflammatory skin conditions, causing scaly and red skin lesions, with predominant effects to areas of high sebum production like the scalp, face, trunk, and skin folds.^1^ Typically, the initial treatments for SD are topical antifungals or corticosteroids. However, these often lead to frequent relapses and patients may struggle with adhering to these treatments, especially in severe or resistant cases.^2^, ^3^ Extended use of topical corticosteroids can also result in complications such as skin thinning, perioral dermatitis, and enlarged blood vessels on the skin.^4^ The challenges in patient compliance and the time‐intensive nature of these topical therapies have prompted the exploration of alternative treatment methods for SD.

Treatment options for SD depend on its severity. Topical treatments such as mild‐to‐moderate corticosteroids, ciclopirox, ketoconazole, miconazole (antifungals), and propylene glycol (a keratolytic/humectant) are effective for mild‐to‐moderate cases. Meanwhile, topicals including ciclopirox, ketoconazole, clotrimazole, lithium succinate/gluconate, corticosteroids, and topical calcineurin inhibitors are recommended for SD on the face or other body areas.^5^, ^6^ Additionally, systemic antifungal medications, namely terbinafine and itraconazole, along with UVB phototherapy, may be considered for severe or resistant cases.^7^ In particularly stubborn cases, healthcare providers may prescribe other specific topicals and systemic medications combined with UVB phototherapy.^8^

Exosome therapy, a cell‐free treatment approach, is increasingly recognized for its potential due to the diminutive size, natural derivation, lipid bilayer structure, and capacity for influencing molecular processes of exosomes.^9^, ^10^ The study of exosomes has shown promise in immunomodulation, contributing to wound healing, scar reduction, skin tone improvement, wrinkle treatment, hair regrowth,^11^ and the management of prolonged inflammatory skin conditions such as bullous pemphigoid, systemic lupus erythematosus, and atopic dermatitis (AD).^9^, ^12^, ^13^ Notably, exosomes from adipose‐derived MSCs have been effective in treating facial redness associated with dupilumab in AD patients.^14^ Additionally, combining exosomes with fractional CO_2_ laser treatments for acne scars has led to faster recovery, fewer side effects, and enhanced treatment results.^15^

Herein, we investigated the efficacy of topical application of moisturizer containing exosomes for the inflammatory skin condition improvement in a patient with recalcitrant SD.

## 2 CASE REPORT

A male patient, aged 40 years old, presented with a severe recurrent SD during his teenage years. He had previously received treatment with topical corticosteroids, topical calcineurin inhibitors, oral antihistamines, and anti‐inflammatory moisturizers for a period of 10 years. The treatment only revealed partial response and recurrence. Also, oral prednisolone was occasionally given as a short‐course treatment for his flare‐up conditions.

For the alleviation of SD symptoms, the 2.5 mL exosomes (ASCE + Skin Rejuvenation Lyophilized Vial (SRLV)‐S, ExoCoBio Inc., Seoul, Republic of Korea) was applied. This novel treatment option involved the application of 20 mg lyophilized rose stem cell‐derived exosomes (RSCEs) by using electroporation (Dermashock Cool, Aestech USA California, USA) to the entire face as an exosomes‐enhanced transdermal drug delivery system.

The evaluation of treatment efficacies used the PGA and IGA used a 5‐point scale from 0 (Clear) to 4 (Severe) to rate disease severity, higher scores indicated worse conditions. Scaling was rated on a 4‐point scale from 0 (None: No visible scaling on lesions) to 3 (Severe: Thick, flaky scales shedding into clothes or skin); and erythema was evaluated on a 4‐point scale from 0 (None: No erythema present) to 3 (Severe: Bright fiery red erythema), with higher scores denoting more severe erythema.^16^

There was a significant improvement of clinical manifestations, including symptoms like redness, scaling, and itching within only 1 day after the initial treatment. (Figure 1B). Hence, we prescribed the patient a regimen of post‐procedural treatment for maintenance to prevent flare‐ups of the disease, which included the application of a twice‐daily topical moisturizer with exosomes (Exobalm, ExoCoBio Inc., Seoul, Republic of Korea) to the entire face for 21 days, followed by nightly application for 1 month, and then maintenance with twice‐weekly application for an additional month. The moisturizer contained RSCEs of 2.5 billion‐particle lyophilized capsules, growth factors, peptides, vitamins, and minerals, combined with a cream of tranexamic acid, madecassoside, and d‐panthenol niacinamide mixtures. Throughout the six‐month follow‐up period, the patient remained free from disease flare‐ups and did not require any additional treatments, as depicted in Figure 1A–C. However, during the sixth month of follow‐up, the patient experienced a flare‐up of seborrheic dermatitis (SD), which was attributed to a lack of sleep and exposure to particulate matter 2.5 (PM2.5) environmental triggers. Intriguingly, this recurrence of the condition presented with a longer duration of symptom‐free remission and less severity compared to the previous flare‐up from the clinical evaluation (Figures 1D and 2).

**FIGURE 1:**
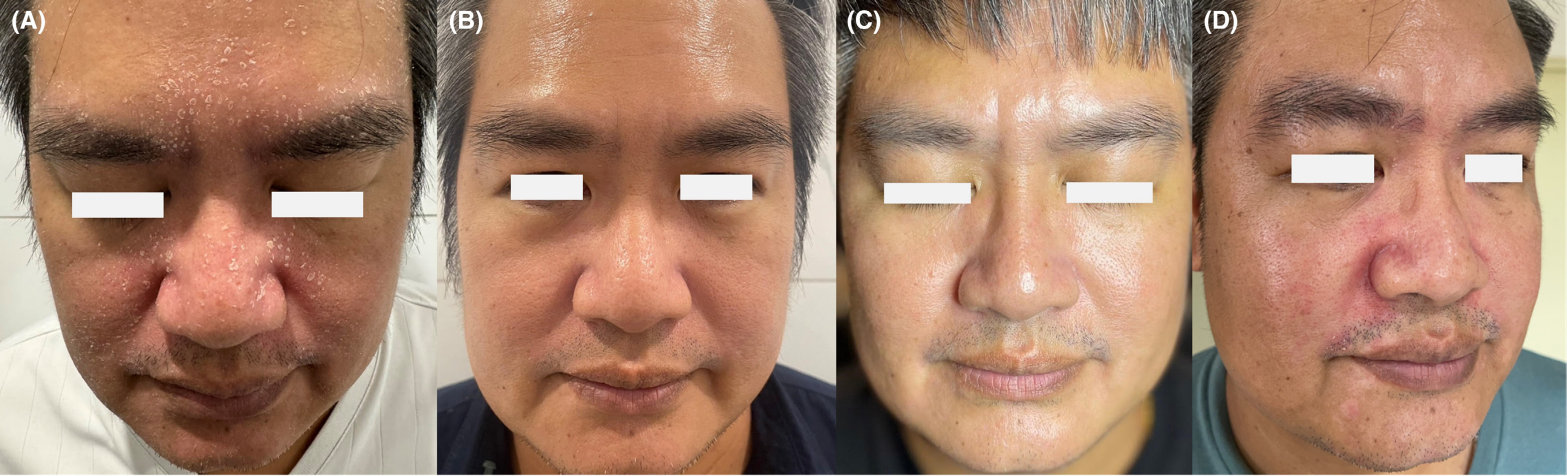
(A) Seborrheic dermatitis flare‐up before treatment, (B) improvement of SD symptoms at Day 2 after treatment, (C) sustained improvement at Month 6 after treatment, (D) the flare‐up after 6 months.

**FIGURE 2:**
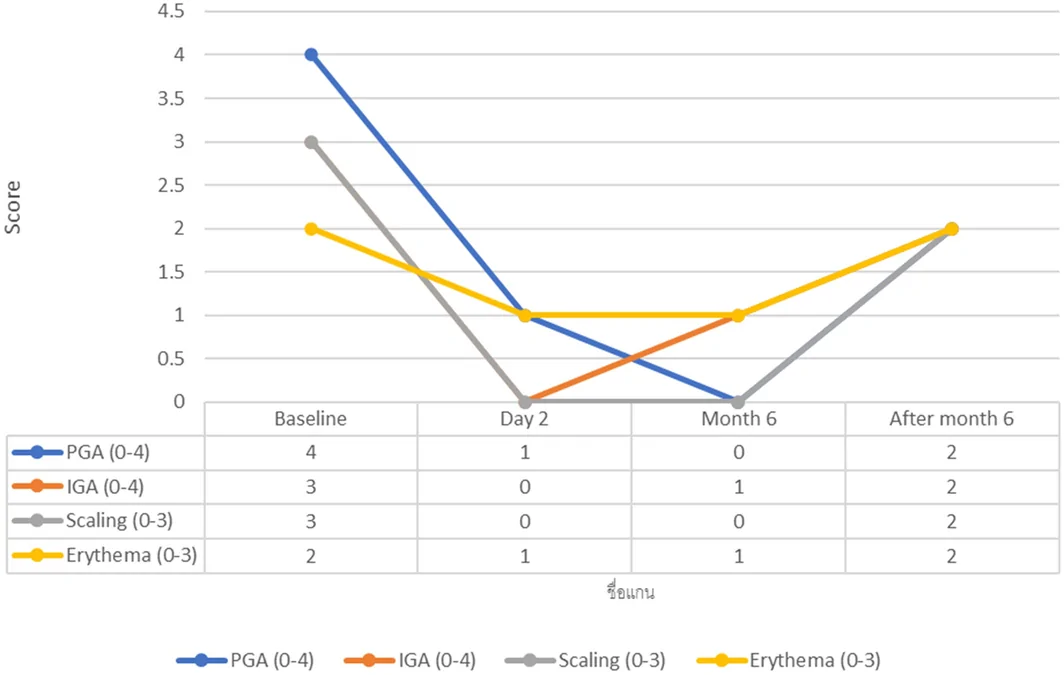
The clinical evaluation using Patient's Global Assessment score (PGA), Investigator's Global Assessment (IGA) score, scaling score, erythema score at baseline, Day 2, Month 6, and after Month 6, respectively.

## 3 DISCUSSION

SD is an inflammatory skin condition with common effects to the face, scalp, and chest. Patients with persistent, recurrent, and treatment‐resistant cases often experience a significant decline in their quality of life.^7^ This condition can profoundly impact both physical and emotional well‐being, causing discomfort, social stigma, lowered self‐confidence, and limited social activities.^7^ Even in milder, symptom‐free forms, SD can result in decreased self‐confidence and a negative social image, particularly among young individuals, women, and those with scalp involvement.^17^

Therefore, this report introduces an alternative treatment approach involving a topical exosome‐containing moisturizer for SD. This option is particularly relevant for cases that do not respond adequately or experience recurrence after standard treatments. The goal is to regulate and diminish the production of sebum and colonization of skin by *Malassezia* spp., in line with inflammation management.^1^, ^8^ While topical corticosteroid treatment is a commonly employed therapeutic method for SD, its long‐term use can cause adverse effects, including skin atrophy, perioral dermatitis, or telangiectasia.^8^

Exosome treatment is a recent cell‐free therapeutic development. Following the small size of exosomes, in line with its biological origins and lipid bilayer membranes, these properties could enhance intracellular communication and capacity of molecular activities to recipient cells. According to the exosome study, there were high immunomodulators for treatment effects of exosomes, for example wound healing, scar prevention, skin pigmentation, wrinkles,^13^ and prolonged inflammatory skin disorders improvement.^12^, ^13^ A study on topical application of human adipose tissue‐derived mesenchymal stem cell (MSC) and ‐derived exosomes (ASCEs) revealed treatment efficacy of dupilumab‐related facial redness (DFR) among AD patients.^14^ Besides, a shorter recovery time was observed from ASCEs with less side effects and better treatment outcomes when combined with fractional CO_2_ laser for acne scars.^15^ In another study, the exosome‐like nanoparticles derived from rose stem cell culture media was isolated by the proprietary extracellular vehicles (EVs) isolation technology for RSCEs profile characterization with multiple skin biological effects, such as RSC in culture‐released RSCEs. In the skin‐related assays of RSCEs, there are microRNA and proteins of multiple biological functions, for example the growth of fibroblast and melanin content in melanocytes. Also, RSCEs enhances exosome‐like nanoparticle production, other than its anti‐inflammatory function, by the stimulation of mesenchymal stem cells for skin quality improvement.^10^

For the novel treatment option, the moisturizer with RSCEs^18^ yielded an improvement immediately after the first treatment sessions with anti‐inflammatory effects and epidermal barrier restoration. It could be associated with the contents of RSCEs, such as microRNA and proteins, to induce higher anti‐inflammatory factors.^19^ Nonetheless, the efficacy of topical moisturizer is related to exosomes and other active ingredients for inflammatory skin conditions, for example skin barrier repair property (sebum, hyaluronic acid,^20^ squalene, and ceramide), anti‐inflammation (madecassoside and niacinamide), better wound healing (D‐panthenol), and whitening effects (niacinamide, hydroxyphenyl propamidobenzoic acid and tranexamic acid).

A treatment modality is required for the chronic and relapsing nature of SD towards the effective management. For instance, patients with refractory to topicals are satisfied with oral pulse therapy and experience frequent relapses or a disease affecting a large area.^21^ Moreover, the twice weekly use of 2% ketoconazole shampoo for 4 weeks is recommended for SD treatment. Meanwhile, the intermittent use or once weekly for 6 months could effectively prevent SD of the scalp.^22^ Hence, this case report elucidates the efficient use of a moisturizer with exosomes for the treatment and prevention of SD relapses.

However, this report is limited by number of cases for generalization. Larger, well‐controlled, prospective, randomized studies should be thus recommended to warrant further elucidation of potential treatment of inflammatory skin conditions by using RSCEs moisturizer combined with the novel technology of exosomes.

## 4 CONCLUSION

SD poses challenges due to its chronic nature. This case report explored a novel approach using a topical exosome‐containing moisturizer for SD treatment, including its anti‐inflammatory characteristics and restoration ability of optimal epidermal barrier functions. Results were promising, with rapid improvements and extended remission during the 6‐month follow‐up, despite a minor flare‐up. Exosomes has shown potential in various skin applications, including inflammation control and barrier restoration. While this report offers encouraging results, its small sample size and limitations necessitate larger, controlled studies to fully assess exosome‐containing moisturizers' therapeutic potential in SD.

## FUNDING INFORMATION

No funding was provided for this article.

## CONFLICT OF INTEREST STATEMENT

The authors report no conflict of interest.

## ETHICS STATEMENT

All procedures performed in studies involving human participants were in accordance with the ethical standards of the 1964 Helsinki Declaration and its later amendments or comparable ethical standards. Informed consent was obtained from the participants in the study.

## PATIENT CONSENT

The patient signed the informed consent form after understanding the nature of the trial.

## Data Availability

The data that support the findings of this study are available on request from the corresponding author. The data are not publicly available due to privacy or ethical restrictions.
